# Altered landscape of total RNA, tRNA and sncRNA modifications in the liver and spleen of mice infected by *Toxoplasma gondii*

**DOI:** 10.1371/journal.pntd.0012281

**Published:** 2024-06-21

**Authors:** Xiao-Xuan Zhang, Yu-Zhe Sun, Wei Wang, Yang Gao, Xin-Yu Wei, Hong-Chao Sun, Chun-Ren Wang, Hong-Bo Ni, Xing Yang, Hany M. Elsheikha, Huan-Ping Guo

**Affiliations:** 1 College of Veterinary Medicine, Qingdao Agricultural University, Qingdao, PR China; 2 State Key Laboratory for Animal Disease Control and Prevention, Harbin Veterinary Research Institute, Chinese Academy of Agricultural Sciences (CAAS), Harbin, China; 3 College of Animal Science and Veterinary Medicine, Heilongjiang Bayi Agriculture University, Daqing, PR China; 4 Institute of Animal Husbandry and Veterinary Medicine, Zhejiang Academy of Agricultural Science, Hangzhou, PR China; 5 Department of Medical Microbiology and Immunology, School of Basic Medicine, Dali University, Dali, PR China; 6 Faculty of Medicine and Health Sciences, School of Veterinary Medicine and Science, University of Nottingham, Sutton Bonington Campus, Loughborough, United Kingdom; Universidade Federal de Uberlandia, BRAZIL

## Abstract

**Background:**

Pathogens can impact host RNA modification machinery to establish a favorable cellular environment for their replication. In the present study, we investigated the effect of *Toxoplasma gondii* infection on host RNA modification profiles and explored how these modifications may influence the host-parasite interaction.

**Methodology/principal findings:**

We analyzed the modification levels of ∼ 80 nt tRNA and 17–50 nt sncRNAs in mouse liver, spleen, and serum using liquid chromatography and tandem mass spectrometry analysis. The results revealed alterations in RNA modification profiles, particularly during acute infection. The liver exhibited more differentially abundant RNA modifications than the spleen. RNA modification levels in serum were mostly downregulated during acute infection compared to control mice. Correlations were detected between different RNA modifications in the liver and spleen during infection and between several RNA modifications and many cytokines. Alterations in RNA modifications affected tRNA stability and protein translation.

**Conclusions/significance:**

These findings provide new insight into the role of RNA modifications in mediating the murine host response to *T. gondii* infection.

## Author summary

The dynamic landscape of host tissue RNA modifications and its possible association with the immune response to *Toxoplasma gondii* infection are poorly understood. Here, we analyzed the patterns of 20 RNA modification types in the liver, spleen and serum of mice infected by *T. gondii*. We characterized the levels of modifications of total RNA, ∼ 80 nt tRNA and 17–50 nt sncRNAs using LC-MS/MS. Differentially abundant RNA modifications were more frequent during acute infection compared to chronic infection and were more pronounced in the liver than in the spleen. Multiple tRNA modifications were co-regulated during chronic infection. Several RNA modifications showed significant correlation with serum cytokines, highlighting the role of RNA modifications in the immune response to *T. gondii* infection. Alterations of RNA modifications affected tRNA stability and the translation of eukaryotic initiation factor 4A/E. These findings provide new insight into the role of RNA modifications in the pathogenesis of and immune response to *T. gondii* infection.

## Introduction

*Toxoplasma gondii* is an obligate intracellular apicomplexan protozoan with a worldwide distribution and a substantial public health impact [1]. This parasite cycles between a felid definitive host and a wide range of vertebrate warm-blooded animals as intermediate hosts. It can also infect humans through the ingestion of raw meat containing the parasite tissue cysts or by drinking of water contaminated with the oocysts excreted in cat feces [2]. While generally harmless, *T. gondii* can cause serious disease in immunocompromised individuals, such as transplant patients and people living with HIV/AIDS [2] as well as in infants congenitally infected during pregnancy [3,4].

Entirely dependent on the host cell machinery for replication, *T. gondii* is known to co-opt host molecules and pathways for its benefit [5]. Previous transcriptomic [6–8], proteomic [9,10], and metabolomic [11,12] studies identified many host genes, proteins and metabolites whose expression is altered during infection, playing key roles in pathways (e.g. immune-metabolic) regulating various aspects of the host-parasite interaction [6,10,13,14]. These studies suggest a complex interaction between *T. gondii* and its host, highlighting the need for more understanding to develop better control strategies for *T. gondii* infection.

RNA modifications play key roles in various biological and pathological processes [15–17]. They are vital players in regulating gene expression in response to a dynamic cellular microenvironment. Over 170 different types of enzymatic RNA modifications have been identified in eukaryotes, many of which are critical for maintaining RNA stability and optimizing its activities and functions [18,19]. In a previous study, we elucidated strain-specific differences in the levels of tRNA and 17–50 nt sncRNA modifications in *T. gondii*, detecting significant correlations between multiple RNA modifications and mRNA expression of some virulence factors, highlighting RNA modifications’ role in parasite pathogenicity [20]. Other studies have shown that *N*^*6*^-methyladenosine (m^6^A) promotes *T. gondii* replication [21] and bradyzoite differentiation [22]. However, the RNA modification profiles of host tissues during *T. gondii* infection and their potential roles in influencing the host response are poorly understood.

The liver and spleen, as major immune organs, play key roles in mediating the host immune response to infection. Unravelling the epitranscriptomic modifications in these tissues during *T. gondii* infection can improve or understanding of the underlying immune-regulatory processes. Herein, we used liquid chromatography and tandem mass spectrometry (LC-MS/MS) to detect the spectrum of RNA modifications in the liver, spleen, and serum of BALB/c mice during the acute and chronic stages of *T. gondii* infection. We explored the possible roles of RNA modifications in the host immune response and analyzed the expression of several cytokines in the spleen. Our results provide a comprehensive profile of RNA modifications in key mouse tissues infected by *T. gondii*.

## Methods

### Ethics statement

All animal experiments were conducted under a protocol approved by the Animal Research Ethics Committee of Qingdao Agricultural University (Approval number: RECQAU201935).

### Animals

Female, 8-10-week-old, BALB/c mice were purchased from Spaefer Biotechnology Company (Beijing, China). All the mice were housed under a 12-h dark/light cycle with free access to food and water at the animal facility of Qingdao Agricultural University. After one week of acclimatization to the facility’s environment, the mice (*n* = 12) were randomly allocated into two groups: a control group (*n* = 6) and an infection group *(n* = 6). The mice in the infection group were infected with 20 *T. gondii* PRU (type I) strain cysts in 1 ml phosphate buffered saline (PBS), while those in the control group were treated with an equal volume of PBS only. At 11 and 33 days post infection (dpi), three mice from each group were euthanized. These time points correspond to the time required for the development of acute and chronic *T. gondii* infection in mice. Following euthanasia, the liver, spleen, and blood serum were immediately collected. The spleen and liver were stored in liquid nitrogen and the serum samples were stored at -80°C. The establishment of *T. gondii* infection was confirmed by PCR as previously described [23].

### Extraction of total RNA from liver and spleen

Total RNA was extracted from the liver and spleen using TRIzol (Life Technologies, Carlsbad, USA) according to the manufacturer’s protocol. Briefly 1 ml TRIzol was added to a 1.5 ml microtube containing pulverized tissues and vigorously vortexed. Then, 200 μl chloroform was added to the sample, which was vortexed and incubated for 10 min at room temperature, followed by centrifugation at 12,000 × g for 15 min at 4°C. The supernatant was transferred to a new 1.5 ml microtube and mixed with an equal volume of isopropanol. After gentle mixing, the mixture was incubated for 10 min at room temperature, followed by centrifugation as described above. The total RNA pellet was resuspended in RNase-free water and stored at -80°C for further use. The quantity and purity of the isolated RNA were determined using a NanoDrop spectrophotometer (ND-1000; Thermo Scientific, DE, USA).

### Isolation of ∼ 80 nt and 17–50 nt RNA fragments from total RNA of liver and spleen

Total RNA isolated from the liver and spleen was separated on a 15% denaturing polyacrylamide gel electrophoresis (PAGE) gel, and different RNA fragments were isolated. Briefly, 4 μg of total RNA was loaded onto the gel and electrophoresed for 1 h in 1× Tris-Borate-EDTA buffer (TBE) buffer at 200 V. The gel was stained by SYBR GOLD (Invitrogen, Waltham, MA, USA). The ∼ 80 nt and 17–50 nt RNA fragments were excised and extracted from the gel using a single-stranded RNA marker-based gel excision approach according to the RNA marker kit (NEB, Ipswich, MA, USA) as previously described [24]. The isolated specific RNA fragments included a mixture of RNAs with a similar range of nucleotide lengths. The quantified level of RNA modifications reflects the total RNA modification levels in the different RNAs contained in each RNA group. In a previous study, ∼ 80 nt RNA isolated from mouse tissue was considered enriched in tRNAs [25]. Therefore, we considered ∼ 80 nt RNA fragments as tRNA here. In another study, PANDORA-seq (an improved sncRNA sequencing method that expands the repertoire of sncRNAs by overcoming RNA modification) was used to characterize 15–50 nt sncRNAs in mouse tissues, which were found enriched in rRNA-derived small RNAs (rsRNAs), tsRNAs, miRNAs, piRNAs, and YRNA-derived small RNAs (ysRNAs) [26]. Therefore, it is reasonable to assume that the 17–50 nt sncRNAs isolated in the present study are mainly composed of the aforementioned sncRNAs.

### Extraction of total RNA from serum

Total RNA was extracted from the serum using TRIzol LS reagent (Invitrogen, Waltham, MA, USA) according to the manufacturer’s protocol. Briefly, serum was added to a microtube, mixed with 3 volume of TRIzol LS reagent, and vigorously vortexed. The mixture was incubated for 5 min at room temperature. Then, 1/5 volume chloroform was added to the mixture, followed by vortexing. After incubation for 10 min at room temperature, the samples were centrifuged at 12,000 × g for 15 min at 4°C. The supernatant was transferred to another microtube, mixed with an equal volume of isopropanol, and stored at -80°C for 30 min to precipitate the RNA. After centrifugation at 12,000 × g for 30 min at 4°C, the pellet was washed with 75% ethanol. Finally, the RNA pellet was suspended in RNase-free water and stored at -80°C until further use.

### Validation of expression using quantitative (q)RT-PCR

Quantitative reverse transcription PCR (qRT-PCR) was used to validate the expression of key cytokines, including interleukin (IL)-2, IL-4, IL-5, IL-6, IL-12, tumor necrosis factor-beta (TNF-β), and interferon gamma (IFN-γ) in mouse spleen. The total RNA samples extracted from the spleen were treated with DNase I (RQ1 RNase-free DNase-Promega, USA) to remove genome DNA. RNA was then used to synthesize cDNA using the M-MuLV Reverse Transcriptase Reaction system (NEB, Ipswich, MA, USA) with a common primer. The obtained cDNA was diluted three times for quantitative PCR (qPCR). All the primers used in the study are listed in Table 1. We used SYBR Green (Promega, USA) for detection of gene expression on a LightCycler 480, according to the manufacturer’s instructions. The relative fold change was calculated using the 2^−ΔΔCT^ method, with actin was used as a reference gene to normalize the relative expression levels.

**Table 1:** Primer sequences used in RT-PCR analysis.

Target gene	Oligonucleotide sequences (5’- 3’)	
*Actin*	F: GGAGATTACTGCCCTGGCTCCTA	R: GACTCATCGTACTCCTGCTTGCTG
*IL-2*	F: GGAACCTGAAACTCCCCAGG	R: AATCCAGAACATGCCGCAGA
*IL-4*	F: CCATATCCACGGATGCGACA	R: AAGCCCGAAAGAGTCTCTGC
*IL-5*	F: CGTGGGGGTACTGTGGAAAT	R: AATCCAGGAACTGCCTCGTC
*IL-6*	F: GCCTTCTTGGGACTGATGCT	R: TGTGACTCCAGCTTATCTCTTGG
*IL-12*	F: AGGACTCACCAGAAGCAAGC	R: CACCCTGTTGATGGTCACGA
*INF-γ*	F: GTAGCCTCACCGCCTATCAC	R: GGGCCTCTCCTGTGAGTCTA
*INF-β*	F: AAGCTCCTCAGCGAGGACAG	R: CGCGGATCATGCTTTCTGTG

### Quantitative analysis of RNA modifications by LC-MS/MS

Total RNA and various types of RNA fragments were digested into mononucleotides as described previously [27,28]. Briefly, RNAs were digested in a 30 μl reaction system containing 3 μl of 10× RNA hydrolysis buffer (2500 mM Tris-HCl, pH 8.0; 50 mM MgCl_2_; and 5 mg/mL BSA), 1 IU benzonase (Sigma-Aldrich, St Louis, MO, USA), 0.2 IU alkaline phosphatase (Sigma-Aldrich, St. Louis, MO, USA), and 0.05 IU phosphodiesterase I (Thermo Fisher Scientific, Grand Island, NY, USA) at 37°C for 3 h. The enzymes in the digestion mixture were then removed using a Nanosep 3K spin filter (Pall Corporation, Ann Arbor, MI, USA) for subsequent liquid chromatography and tandem mass spectrometry (LC-MS/MS) analysis. The abundance of each RNA modification was quantified according to a standard curve and established RNA modification relative quantification methods [27,28]. The modified nucleotide standards used in this study are listed in S1A Fig. The percentage of each modified ribonucleoside was normalized to the total amount (molar concentration) of all the quantified ribonucleosides detected.

### Northern blot analysis

Northern blotting was performed to evaluate tRNA expression levels in the mouse liver during *T. gondii* infection as previously described [29]. Total RNA was separated on a 15% urea-PAGE gel. The gel was stained, imaged, and immediately transferred onto Roche Nylon Membranes (Roche, Basel, Switzerland) and cross-linked using UV. The membrane was prehybridized using Roche DIG hybridization buffer (Roche, Basel, Switzerland). For detection of tRNA^Ala^, tRNA^Gly^, tRNA^Val^ and tRNA^Leu^, the membrane was incubated overnight at 42°C with DIG labeled probes (5’-tRNA^Leu^: 5’-DIG-CCTTAGACCGCTCGGCCATCCTGAC; 5’-tRNA^Ala^: 5’-DIG-CGCTCTACCACTGAGCTACACCCCC; 5’-tRNA^Val^: 5’-DIG-GTGATAACCACTACACTACGGAAAC; 5’-tRNA^Gly^: 5’-DIG-AATTCTACCACTGAACCACCCATGC). After 24 h, the membrane was washed with a low stringency buffer (2 × SSC with 0.1% [wt/vol] SDS), followed by two washes with a high stringency buffer (0.1 × SSC with 0.1% [wt/vol] SDS) and washing buffer (1 × SSC). The membrane was then incubated with blocking buffer (Roche, Basel, Switzerland) at room temperature for 3 h, followed by incubation in a blocking buffer with anti-Digoxigenin-AP Fab fragment (Roche, Basel, Switzerland) diluted 1:10,000. After further incubation in developing buffer at room temperature, the membrane was coated with CSPD (Roche, Basel, Switzerland) for 15 min at 37°C in the dark and imaged using a Bio-Rad system (USA).

### Western blot analysis

Western blotting was performed to evaluate the expression levels of eukaryotic initiation factors 4A (eIF4A) and 4E (eIF4E) in the liver of mice infected by *T. gondii* for 11 days. Briefly, lysates from the liver were mixed with 5× SDS gel loading buffer, denatured, and separated by SDS-PAGE. Primary antibodies used were: Beta Actin Mouse McAb (Proteintech, Chicago, USA), eIF4A (C32B4) rabbit mAb (CST, Massachusetts, USA), and eIF4E (CST, Massachusetts, USA). Horseradish peroxidase (HRP)-conjugated goat anti-rabbit immunoglobulin G (Proteintech, Chicago, USA) and HRP-conjugated goat anti-mouse immunoglobulin G (Beyotime, Shanghai, China) were used as secondary antibodies. The blots were scanned using a Bio-Rad system (USA). The mean values of eIF4A and eIF4E amounts and standard deviations were calculated from three biological replicates.

### Statistical analysis

All statistical analyses were performed using GraphPad Prism 8 (GraphPad Software Inc, San Diego, CA, USA). The unpaired Student’s *t*-test and Pearson’s correlation analysis were performed to examine the correlations between RNA modifications and the expression levels of the RNA-modifying methyltransferase enzymes or cytokines. All results represent the means + standard error of the mean (SEM), and a *p*-value of < 0.05 was considered statistically significant.

## Results

### Acute *T. gondii* infection causes extensive alterations in RNA modifications

**Fig 1:**
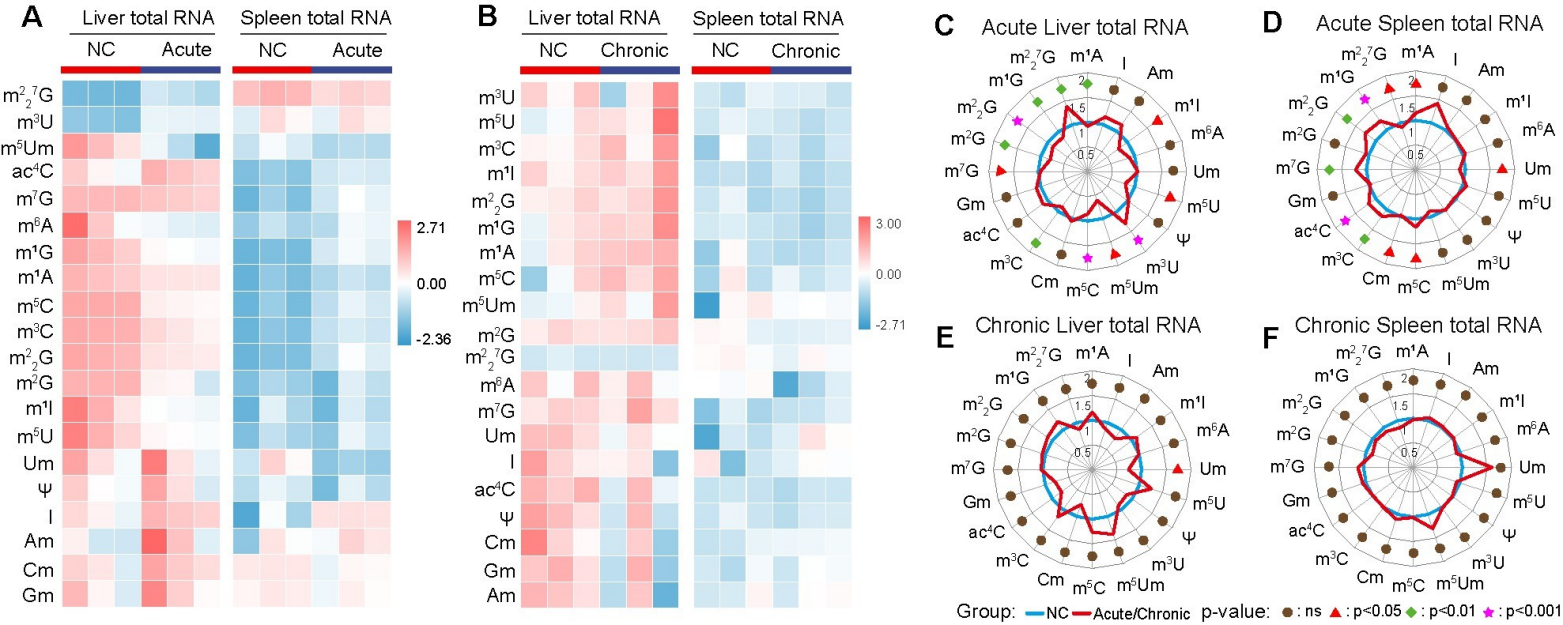
*Toxoplasma gondii* infection induces RNA modifications in the liver and spleen total RNA of mice. (**A-B**) Heatmaps showing RNA modifications in the liver and spleen in the control group (NC) versus acutely infected (A) and chronically infected groups (**B**) (*n* = 3/group). Color values represent log10 (percentage of RNA modification levels). (**C-D**) Radar charts displaying RNA modifications in the liver (**C**) and spleen (**D**) between control and acutely infected groups. (**E-F**) Radar charts showing RNA modifications in the liver (**E**) and spleen (**F**) between control and chronically infected groups. The average RNA modification level in the NC group was set to 1.

To determine the impact of *T. gondii* infection on the host epitranscriptome, we performed LC-MS/MS analysis on liver, spleen, and serum samples from mice infected by *T. gondii* for 11 or 33 days, comparing them to uninfected mice. We quantified 20 types of RNA modifications and four unmodified ribonucleosides (A, U, C and G) in the total RNA (S1B Fig). The liver and spleen showed distinct RNA modification signatures, with the liver exhibiting a higher relative abundance of most RNA modifications compared to the spleen (Fig 1A and 1B). These RNA modifications displayed different expression patterns between the liver and spleen and between acute and chronic infections. We detected 12 and 10 differentially abundant RNA modifications in the liver and spleen, respectively (Fig 1C and 1D). In acute infection, 10 types of RNA modifications (m^5^C, m^2^_2_G, m^2^G, m^3^C, m^1^G, m^1^A, m^1^I, m^5^U, m^5^Um, m^7^G) were significantly decreased in the liver, while two RNA modification types (m^2^_2_7G, *p* < 0.01, and m^3^U, *p* < 0.0001) were increased compared to control mice (S1C Fig). Conversely, in the spleen of acutely infected mice, three RNA modification types (Um, m^2^_2_^7^G and Cm) were downregulated and seven types (ac4C, m^1^G, m^3^C, m^7^G, m^2^_2_G, m^5^C, m^1^A) were upregulated (S1D Fig). In chronic infection, only Um was significantly decreased in the liver and no distinct RNA modification were deregulated in the spleen (Figs 1C and 1D and S1E). Serum RNA modifications were mostly downregulated during acute infection (I, ac^4^C, Am, *p* < 0.05; m^5^Um, m^5^C, m^7^G, m^6^A, *p* < 0.01; m^1^A, m^2^_2_G, m^1^I, m^3^C, m^1^G, m^2^G; *p* < 0.001) and slightly during chronic infection (m^5^U, Psi, *p* < 0.05), compared to uninfected mice. However, three RNA modifications (Am, Cm, Gm, *p* < 0.05) were upregulated during chronic infection compared to uninfected mice (S2 Fig). Taken together, these results indicate that acute *T. gondii* infection induces more RNA modifications in the liver and spleen total RNA than chronic infection.

### *T. gondii* infection alters tRNA and sncRNA modification levels

**Fig 2:**
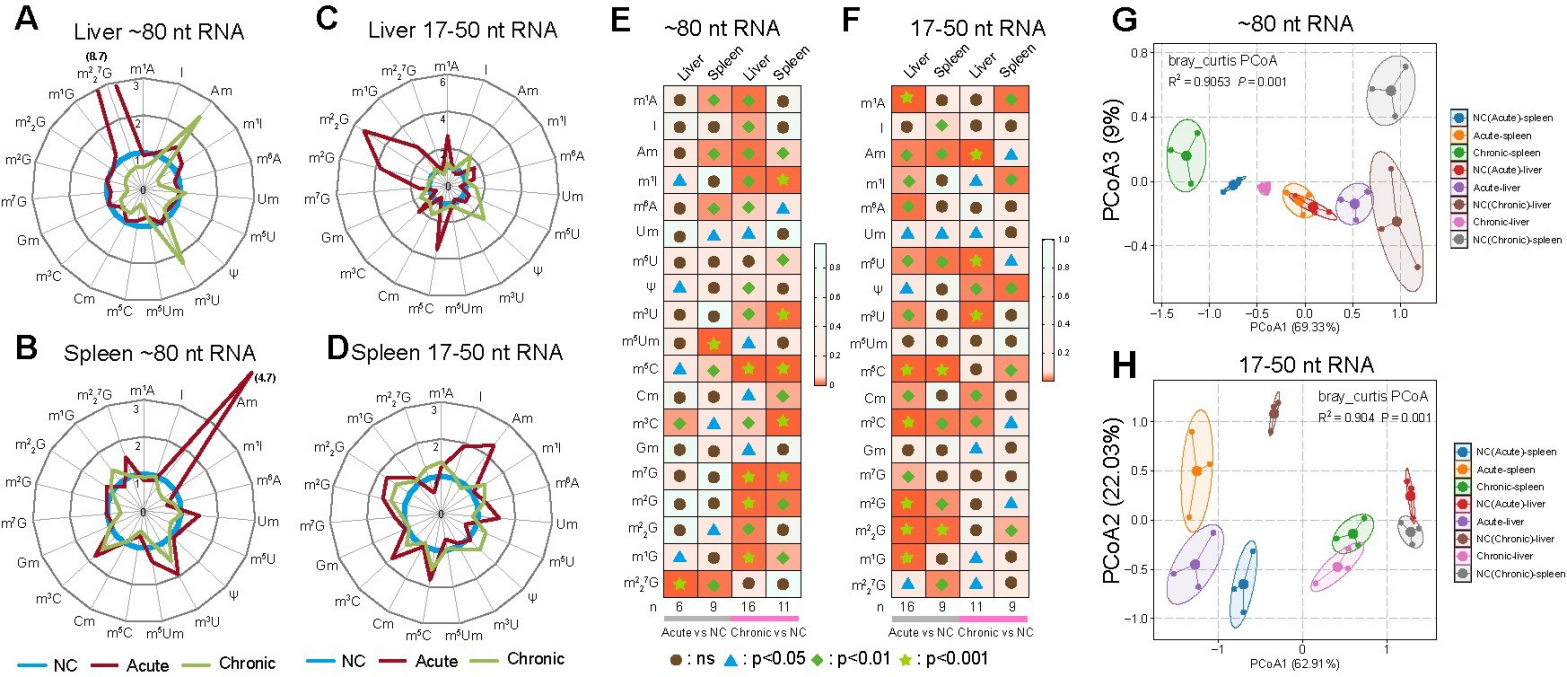
*T. gondii* infection alters tRNA and sncRNA modification signatures in the liver and spleen of mice. (**A-B**) Radar charts showing the relative tRNA modifications in liver (**A**) and spleen (**B**) between control, acute, and chronic infection mouse groups. The average of RNA modification in three repeats was calculated. Then, the level of each RNA modification in the NC group was considered as 1. **(C-D**) Radar charts displaying relative sncRNA modifications in liver (**C**) and spleen (**D**) between control, acute, and chronic infection groups. (**E**) Comparison of tRNA modifications between control (NC) and infection groups across tissues. (**F**) Comparison of sncRNA modifications between control and infection groups across tissues. (**G-H**) Principal component analysis (PCA) of RNA modifications in ∼80 nt RNA and 17–50 nt RNA in the liver and spleen during acute and chronic infection. Eighteen types of RNA modifications were analyzed based on their abundance (*n* = 3/group). Statistical analysis used unpaired student’s *t*-test with three independent replicates.

To identify the specific RNA types responsible for the observed RNA modification changes in the liver and spleen during *T. gondii* infection, we compared the differential expression of 19 RNA modifications in ∼ 80 nt RNA (mainly transfer RNA (tRNA) and 17–50 nt (mostly microRNA (miRNA), some tRNA-derived small RNA (tsRNA) and ribosomal-derived small RNA (rsRNA)) during acute and chronic infection. Fig 2A–2D shows the differences in the abundance of RNA modifications between control, acute, and chronic infected groups. During acute infection, m^2^_2_^7^G and Am exhibited high differential abundance in the liver and spleen ∼80 nt RNA, respectively (Fig 2A and 2B), while m^2^_2_G and Am were more significantly upregulated in liver and spleen 17–50 nt RNA respectively (Fig 2C and 2D). For ∼80 nt RNA, 6 and 9 modifications were significantly changed in the liver and spleen, respectively, during acute infection, whereas 16 and 11 modifications were significantly regulated in the liver and spleen during chronic infection (Fig 2E). Regarding 17–50 nt RNA, 16 and 9 modifications were significantly altered in liver and spleen, respectively, during acute infection, while 11 and 9 types were upregulated, respectively during chronic infection (Fig 2F).

Principal component analysis (PCA) of RNA modifications during *T. gondii* infection showed distinct clusters by tissue type (liver and spleen) and infection stage (acute and chronic) for both 17–50 nt sncRNA (Fig 2G) and ∼80 nt tRNA (Fig 2H). This clear segregation suggests that RNA modification patterns, particularly in the tRNA fraction, retain tissue-specific characteristics even after *T. gondii* infection. These findings corroborate with previous studies showing distinct RNA modification profiles between different tissues and even between regions of the same tissue [30]. Additionally, others have reported tissue-specific RNA modifications in hypoxic mice [25] and correlations of RNA modification patterns with parasite strains of differing virulence [20].

### Linear correlations between different RNA modifications during *T. gondii* infection

**Fig 3:**
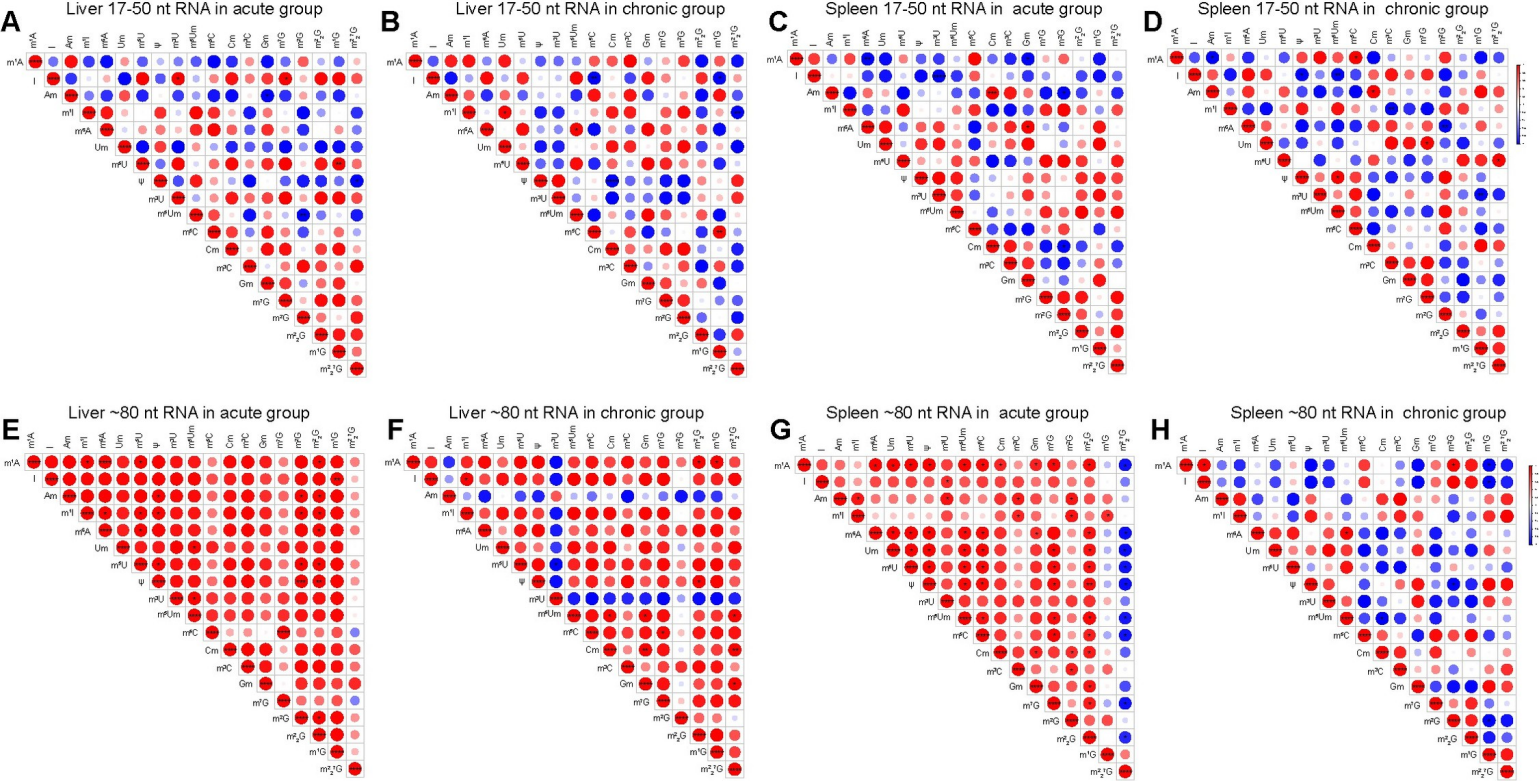
Correlation analysis of RNA modifications in the liver and spleen during *T. gondii* infection. **(A-D)** Pearson correlation analysis of sncRNA modifications in liver during (**A**) acute and (**B**) chronic infection, and spleen during (**C**) acute and (**D**) chronic infection. **(E-H)** Pearson correlation analysis of tRNA modifications in liver during (**E**) acute and (**F**) chronic infection, and spleen during (**G**) acute and (**H**) chronic infection. Correlation coefficients were computed using GraphPad Prism 8, and Pearson *R* values are presented as heatmaps. Significant differences are indicated by asterisk as follows: *p < 0.05, **p < 0.01, ***p < 0.001, ****p < 0.0001.

Coregulation can occur between different RNA modifications. For example, the abundance of sperm tsRNA m^5^C correlates with m^2^G abundance in a high-fat diet-induced metabolic disorder mouse model, and knockout of the m^5^C methyltransferase Dnmt2 decreases both m^5^C and m^2^G levels in sperm tsRNA [24]. We systematically analyzed correlations among RNA modifications in 17–50 nt RNA and ∼80 nt RNA in the liver and spleen during acute *T. gondii* infection. Both positive and negative linear correlations between RNA modifications were comparable in 17–50 nt RNA during acute and chronic infection in liver and spleen (Fig 3A–3D). Interestingly, most of RNA modifications exhibited positive linear correlations in ∼80 nt tRNA in the liver and spleen during acute infection (Fig 3E–3H), especially in the spleen (Fig 3G). During chronic infection, most ∼80 nt tRNA modifications in the liver showed positive linear correlations, whereas spleen tRNA modifications showed negative correlations (Fig 3F and 3H). These results suggest that RNA modifications are involved in the pathogenesis of *T. gondii* infection, with tissue-specific differences in the correlations between different ∼80 nt tRNA modifications during chronic infection.

### RNA modifications correlate with cytokine levels

**Fig 4:**
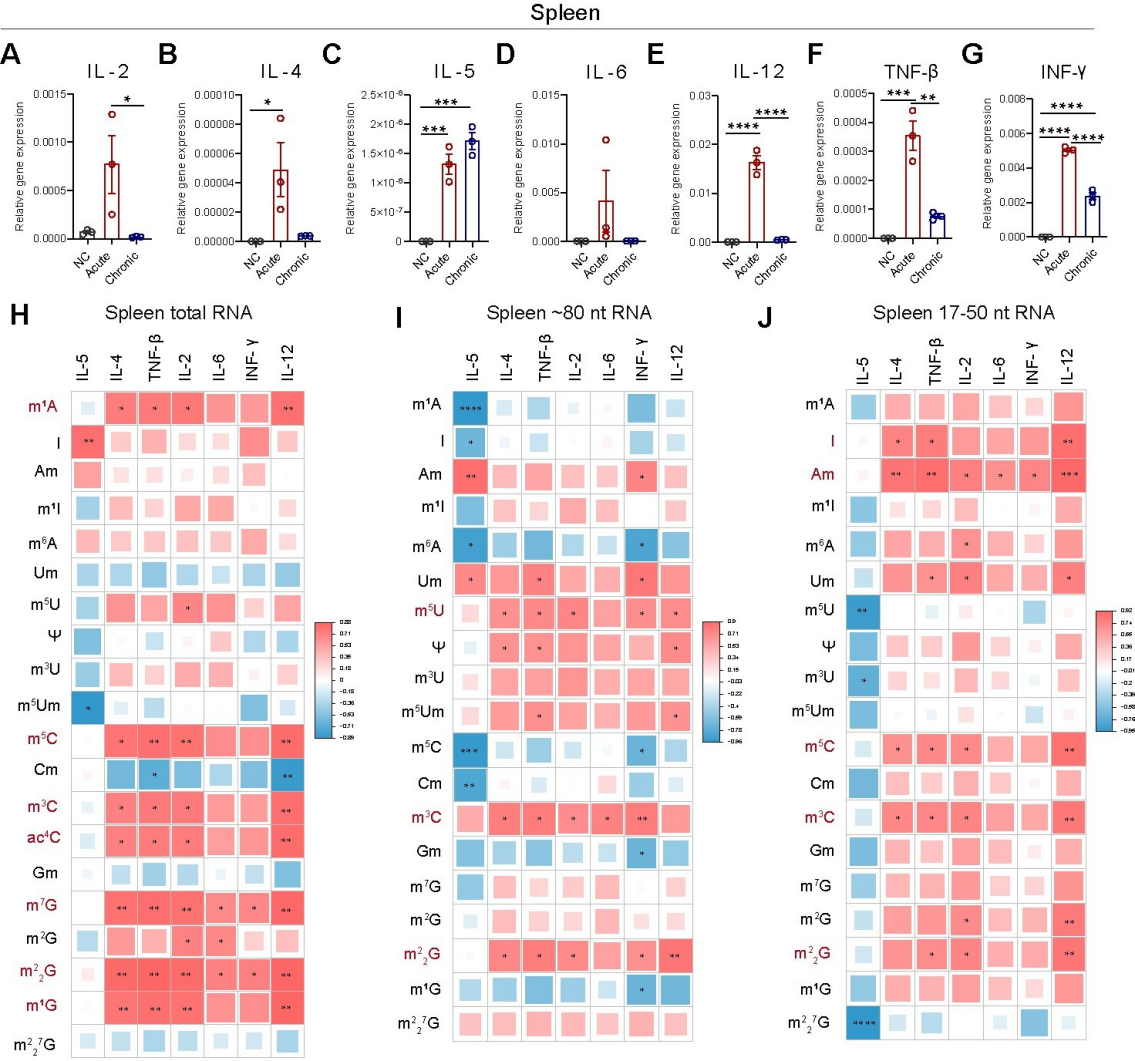
Differential RNA modifications correlate with cytokines. (**A-G**) Comparative analysis of cytokine levels in the spleen among control, acute infection, and chronic infection groups. (**H-J**) Hierarchical clustering and Spearman correlation heat maps showing the linear relationship between modifications levels of (**H**) total RNA, (**I**) tRNA, (**J**) sncRNA, and cytokine expression. RNA modifications exhibiting the strongest correlation with cytokines are shown in red. All data are presented as mean ± SEM. Statistical analysis was performed using one-way ANOVA with uncorrected Fisher’s LSD in panels **A-G**. Significance levels are denoted by asterisk as follows: *p < 0.05, **p < 0.01, ***p < 0.001, ****p < 0.0001.

RNA modifications are closely associated with cytokine expression levels, reflecting the pivotal role of spleen-secreted cytokines in the host response to *T. gondii* infection. We evaluated cytokine expression in the spleen using RNA-seq analysis and confirmed the results via qRT-PCR, demonstrating consistent fold changes between infected and control mice (*p* < 0.0001; *r* = 0.85). Levels of IL-2, IL-4, IL-5, IL-6, IL-12, TNF-β, and INF-γ were significantly elevated in infected mice compared to controls (Fig 4A–4G). Furthermore, cytokine levels were significantly higher in the acute infection group compared to the chronic infection group, with the exception of IL-5 (Fig 4C). To better understand the biological relevance of RNA modifications during *T. gondii* infection, correlations between modification abundance in total RNA, specific fragments, and cytokine levels were examined. We found positive correlations between levels of total RNA modifications (m^1^A, m^5^C, m^3^C, ac^4^C, m^7^G, m^2^_2_G and m^1^G) and cytokine levels (Fig 4H). In the ∼ 80 nt RNA fragments, m^5^U, m^3^C and m^2^_2_G exhibited significant linear correlation with multiple cytokines (Fig 4I). Additionally, levels of sncRNA modifications I, Am, m^5^C, m^3^C and m^2^_2_G correlated with cytokines (Fig 4J).

### Alteration of RNA modification signature in 80 nt RNA affects the tRNA stability

**Fig 5:**
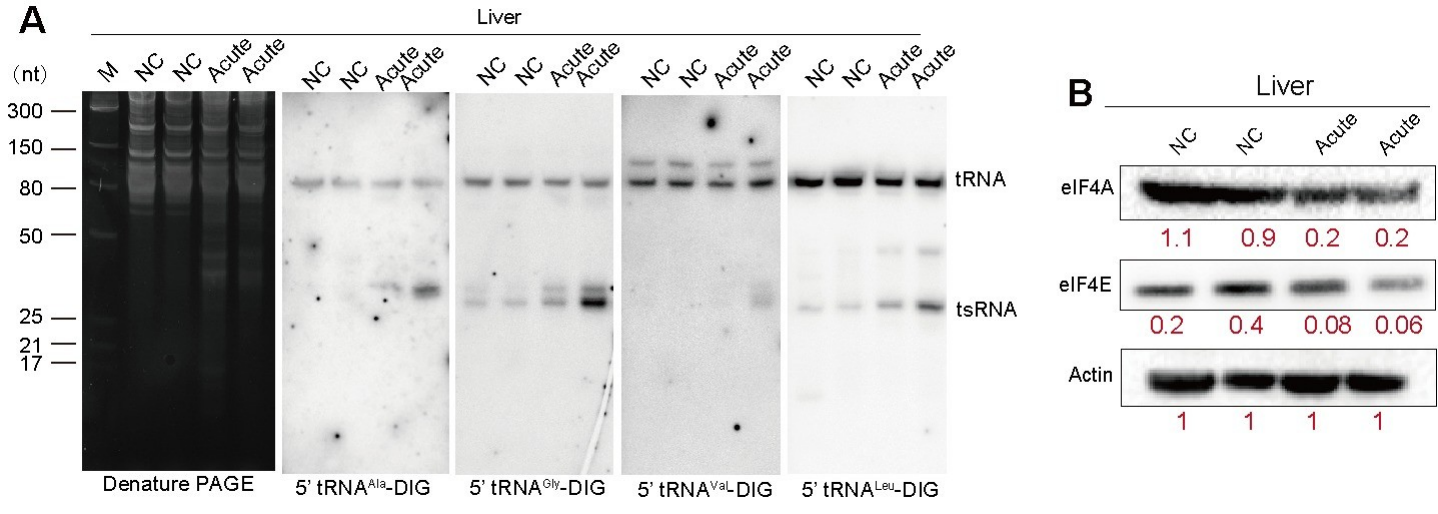
*T. gondii* alters tRNA stability and translation initiation factors expression in the liver during acute infection. **(A)** Northern blot analysis of tRNA^Ala^, tRNA^Gly^, tRNA^Val^ and tRNA^Leu^ in the liver of mice infected by *T. gondii* for 11 days. Data represent mean ± SD from 3 biological replicates, with a representative northern blot membrane shown. **(B)** Western blot analysis of eIF4A and eIF4E levels in the liver of mice infected by *T. gondii* for 11 days. Data represent mean ± SD from 3 biological replicates, with a representative Western blot membrane shown.

To investigate the influence of RNA modifications on tRNA stability, we examined the expression of several tRNAs with high codon usage in mouse proteins. Given the more pronounced change in RNA modification signatures during acute infection compared to chronic infection (Fig 2A and 2B), we assessed the levels of tRNA^Ala^, tRNA^Gly^, tRNA^Val^ and tRNA^Leu^ in the liver during acute infection using northern blotting. The expression levels of these tRNAs decreased, while the corresponding tsRNA levels increased (Fig 5A), suggesting that changes in tRNA modifications during *T. gondii* infection in mice affect the tRNA pool, potentially influencing protein translation efficiency. To examine this assumption, we used Western blotting to measure the expression of eIF4E/A and detected a reduction in their abundance (Fig 5B), indicating an impact on translation initiation, as eIF4E/A are essential for this process.

## Discussion

RNA modifications play important roles in the virulence and infection processes of *T. gondii* [21,22]. However, the changes in RNA modifications within mouse tissues post *T. gondii* infection remain largely unexplored. Here, we provide new insight into the role of epitranscriptomic modifications in the host’s response to *T. gondii* infection. We investigated the abundance of various RNA modifications in the primary immune organs (liver and spleen) and serum of mice infected by *T. gondii* during acute and chronic infection phases. Our analysis revealed that RNA modifications were more prevalent during acute *T. gondii* infection compared to chronic infection, with the liver exhibiting more differentially abundant RNA modifications than the spleen. Additionally, RNA modifications in nt tRNA induced by *T. gondii* infection affected tRNA stability. These results suggest that tRNA and sncRNA modifications are involved in *T. gondii* infection, highlighting the potential roles of RNA modifications in the host response to *T. gondii* infection.

Over 170 chemical modifications have been identified in coding and various noncoding RNAs across all living organisms, including bacteria, archaea, and eukaryotes [18]. However, we quantified only 20 modified RNA nucleobases in mouse tissues due to the limited availability of RNA standards. Single-stranded RNA marker-based gel excision is used to separate different RNA fragments [20,25], and the isolated specific RNA fragments represent a mixture of RNAs with similar nucleotide length ranges. The RNA modification abundance reflects the RNAs contained in each group, potentially affecting the RNA modification profiles of specific RNA classed. For example, sncRNA shares a similar nucleotide length with mature tRNA, and the abundance of RNA modifications on sncRNA may contribute to the alteration of RNA modifications in tRNA post-*T. gondii* infection.

*T. gondii* infection progresses from an acute stage, characterized by rapid replication of tachyzoites, to a chronic stage marked by the formation of persistent dormant cysts [31,32]. A robust Th1-type immune response mediated by pro-inflammatory cytokines, including IFN-γ, TNFα, IL-1β, IL-2, IL6, and IL-12p70, is required to limit *T. gondii* infection during acute infection [33]. During chronic infection, anti-inflammatory cytokines, such as IL-5, restrain excessive inflammatory responses and reduce immunopathological damage to the host [34]. Our results are consistent with previous studies, showing that levels of the pro-inflammatory cytokines IL-2, IL-4, IL-5, IL-6, IL-12, TNF-β, and INF-γ increase during the acute stage, while the level of the anti-inflammatory cytokine IL-5 increases during the acute stage and decreases during the chronic stage [34].

A recent study found that deletion of Nsun2 promotes type I interferon (IFN) response, significantly reducing gene expression and replication of viruses [35]. This m^5^C-mediated antiviral innate immunity is attributed to a decrease in the host m^5^C methylome and the promotion of polymerase III–transcribed noncoding RNAs that are recognized by the cytosolic RNA sensor RIG-I, inducing effective type I IFN signaling [35]. Whether m^5^C tRNA modification plays a role in the innate immune response to *T. gondii* infection by regulating type I interferon remains to be investigated. Nonetheless, the role of RNA modifications in immunity is well-established [36–38]. Intriguingly, positive correlations were detected between the modification levels of total RNA (m^1^A, m^5^C, m^3^C, ac^4^C, m^7^G, m^2^_2_G and m^1^G) and cytokines, and between tRNA (m^5^U, m^3^C and m^2^_2_G) or sncRNA (I, Am, m^5^C, m^3^C and m^2^_2_G), and many cytokines. Most of these RNA modifications play roles in immunity by regulating the biological processes of immune cells [39]. Given these facts, it is reasonable to assume that RNA modifications play a role in regulating the host defense mechanism against *T. gondii* infection during acute infection.

In the present study, levels of tRNA^Ala^, tRNA^Gly^, tRNA^Val^ and tRNA^Leu^ were decreased, while the corresponding levels of tsRNA were increased in the liver during acute infection. This finding agrees with previous studies showing that post-transcriptional modifications in tRNAs influence the regulatory role played by tRNA-derived fragments (tRFs) in stem cells [40] and the pathophysiology of various diseases and pathological conditions [41]. The deposition of tRNA post-transcriptional nucleoside modifications is a dynamic and effective way for cells to regulate and adapt protein translation to external cues, via tsRNAs [16]. A previous study showed that 5’tsRNA^Ala^ displaces the translation initiation factor eIF4E/G/A from m^7^G-capped mRNA, resulting in a reduction in translation [40]. Also, exposure to hypoxia alters the abundance of tRNA modifications in mouse tissues, affecting tRNA stability and translation control [25]. Here, we showed that *T. gondii* reduced the abundance of 5’tsRNA^Ala^ and eIF4E/A, essential for translation initiation, suggesting that altered tRNA modifications by infection can destabilize tRNA and reduce the levels of tsRNA and eIF4E/A, compromising protein translation efficiency.

Changes in tsRNAs may also play a role in modulating the host-pathogen interactions, for example, via immune regulation during infection. A previous study on *Mycobacterium* showed that tRNA and its derived fragments attenuate host immune responses toward *Mycobacterium* by modulating a caspase-8-dependent pathway and inducing apoptosis in host monocytes [42]. tRNA fragments can activate pattern-recognition receptors, such as Toll-like receptors, which play essential roles in the host immune response to infection [43]. The role of tsRNAs in the pathogenesis of *T. gondii* infection, whether through modulating the host immune response, altering genes at the post-transcriptional level, or interfering with intercellular and intracellular communications, remains to be investigated.

In conclusion, our results provide new insight into the epitranscriptomic modifications of the murine tissue in response to *T. gondii* infection, showing the parasite’s ability to cause significant modifications in the liver and spleen ∼ 80 nt tRNAs and 17–50 nt sncRNAs during acute and chronic infection. Correlations were also detected between different types of RNA modifications and between RNA modifications and cytokines. *T. gondii* also decreased the level of 5’tsRNAs and eIF4E/A in the liver during acute infection, influencing protein synthesis and the host cell response to infection. Whether the detected changes in RNA modifications represent an adaptative response to *T. gondii* infection or a passive host cell response to infection-induced stress remains to be elucidated. However, some detected modifications are involved in modulating host immune response mechanisms and cell cycle regulation–mechanisms relevant to the pathogenesis of *T. gondii* infection. This work paves the way for genetic manipulation and more mechanistic insight into the cellular pathways affected by the RNA modifications identified here, such as m^2^_2_^7^G in the liver and Am in the spleen, and their roles in influencing the outcome of *T. gondii* infection.

## Supporting information

S1 Fig Detection of RNA modifications in the RNA of mouse tissues. **(A)** A schematic illustration depicting the experimental procedures used for detecting and quantifying RNA modifications in the spleen, liver, and serum of mice. (**B**) List of the used nucleobase standards. (**C**) Comparison of RNA modifications in total RNA of the liver between the control group and the acute infection group. (**D**) Comparison of RNA modifications in total RNA of the spleen between the control group and the acute infection group. (**E**) Comparison of RNA modifications in the total RNA of liver between the control group and the chronic infection group. All results are shown as mean ± SEM based on three biological replicates. Statistical analysis was conducted using unpaired Student’s *t*-test. Significant differences are indicated by asterisk as follows: *p < 0.05, **p < 0.01, ***p < 0.001, ****p < 0.0001.
(TIF)

S2 Fig The abundance of RNA modifications in the total RNA of the mouse serum. (**A**) Comparison of RNA modifications in the serum total RNA between the control group and the acute infection group. (**B**) Comparison of RNA modifications in the serum total RNA between the control group and the chronic infection group. All results are shown as mean ± SEM based on biological triplicate. Statistical analysis was conducted using unpaired Student’s *t*-test. Significant differences are indicated by asterisk as follows: *p < 0.05, **p < 0.01, ***p < 0.001, ****p < 0.0001.
(TIF)

## References

[1] Montoya JG, Liesenfeld O. Toxoplasmosis. Lancet. 2004;363(9425):1965–76. doi: 10.1016/S0140-6736(04)16412-X 15194258

[2] Elsheikha HM, Marra CM, Zhu XQ. Epidemiology, pathophysiology, diagnosis, and management of cerebral toxoplasmosis. Clin Microbiol Rev. 2021;34(1). doi: 10.1128/CMR.00115-19 33239310 PMC7690944

[3] Elsheikha HM. Congenital toxoplasmosis: priorities for further health promotion action. Public Health. 2008;122(4):335–53. doi: 10.1016/j.puhe.2007.08.009 17964621

[4] Torgerson PR, Mastroiacovo P. The global burden of congenital toxoplasmosis: a systematic review. Bull World Health Organ. 2013;91(7):501–8. doi: 10.2471/BLT.12.111732 23825877 PMC3699792

[5] Rosenberg A, Sibley LD. Toxoplasma gondii secreted effectors co-opt host repressor complexes to inhibit necroptosis. Cell Host Microbe. 2021;29(7):1186–98 e8. doi: 10.1016/j.chom.2021.04.016 34043960 PMC8711274

[6] Wang M, Zhang FK, Elsheikha HM, Zhang NZ, He JJ, Luo JX, et al. Transcriptomic insights into the early host-pathogen interaction of cat intestine with Toxoplasma gondii. Parasit Vectors. 2018;11(1):592. doi: 10.1186/s13071-018-3179-8 30428922 PMC6236892

[7] Wang SS, Zhou CX, Elsheikha HM, He JJ, Zou FC, Zheng WB, et al. Temporal transcriptomic changes in long non-coding RNAs and messenger RNAs involved in the host immune and metabolic response during Toxoplasma gondii lytic cycle. Parasit Vectors. 2022;15(1):22. doi: 10.1186/s13071-021-05140-3 35012632 PMC8750853

[8] Hu RS, He JJ, Elsheikha HM, Zou Y, Ehsan M, Ma QN, et al. Transcriptomic Profiling of mouse brain during acute and chronic infections by Toxoplasma gondii oocysts. Front Microbiol. 2020;11:570903. doi: 10.3389/fmicb.2020.570903 33193165 PMC7604304

[9] He JJ, Ma J, Elsheikha HM, Song HQ, Zhou DH, Zhu XQ. Proteomic profiling of mouse liver following acute Toxoplasma gondii infection. PLoS One. 2016;11(3):e0152022. doi: 10.1371/journal.pone.0152022 27003162 PMC4803215

[10] He JJ, Ma J, Wang JL, Zhang FK, Li JX, Zhai BT, et al. iTRAQ-based quantitative proteomics analysis identifies host pathways modulated during Toxoplasma gondii infection in swine. Microorganisms. 2020;8(4). doi: 10.3390/microorganisms8040518 32260483 PMC7232346

[11] Zhou CX, Zhou DH, Elsheikha HM, Liu GX, Suo X, Zhu XQ. Global metabolomic profiling of mice brains following experimental infection with the cyst-forming Toxoplasma gondii. PLoS One. 2015;10(10):e0139635. doi: 10.1371/journal.pone.0139635 26431205 PMC4592003

[12] Ma J, He JJ, Hou JL, Zhou CX, Elsheikha HM, Zhu XQ. Ultra performance liquid chromatography-tandem mass spectrometry-based metabolomics reveals metabolic alterations in the mouse cerebellum during Toxoplasma gondii infection. Front Microbiol. 2020;11:1555. doi: 10.3389/fmicb.2020.01555 32765450 PMC7381283

[13] Chen XQ, Elsheikha HM, Hu RS, Hu GX, Guo SL, Zhou CX, et al. Hepatic metabolomics investigation in acute and chronic murine toxoplasmosis. Front Cell Infect Microbiol. 2018;8:189. doi: 10.3389/fcimb.2018.00189 29922602 PMC5996072

[14] Ma J, He JJ, Hou JL, Zhou CX, Zhang FK, Elsheikha HM, et al. Metabolomic signature of mouse cerebral cortex following Toxoplasma gondii infection. Parasit Vectors. 2019;12(1):373. doi: 10.1186/s13071-019-3623-4 31358041 PMC6664753

[15] Roundtree IA, Evans ME, Pan T, He C. Dynamic RNA modifications in gene expression regulation. Cell. 2017;169(7):1187–200. doi: 10.1016/j.cell.2017.05.045 28622506 PMC5657247

[16] Frye M, Harada BT, Behm M, He C. RNA modifications modulate gene expression during development. Science. 2018;361(6409):1346–9. doi: 10.1126/science.aau1646 30262497 PMC6436390

[17] Wiener D, Schwartz S. The epitranscriptome beyond m(6)A. Nat Rev Genet. 2021;22(2):119–31. doi: 10.1038/s41576-020-00295-8 33188361

[18] Boccaletto P, Machnicka MA, Purta E, Piatkowski P, Baginski B, Wirecki TK, et al. MODOMICS: a database of RNA modification pathways. 2017 update. Nucleic Acids Res. 2018;46(D1):D303–D7. doi: 10.1093/nar/gkx1030 29106616 PMC5753262

[19] Nachtergaele S, He C. Chemical modifications in the life of an mRNA transcript. Annu Rev Genet. 2018;52:349–72. doi: 10.1146/annurev-genet-120417-031522 30230927 PMC6436393

[20] Wang W, Yang Y, Guo H, Li MH, Chen XQ, Wei XY, et al. Unravelling strain-specific modifications of Toxoplasma gondii tRNA and sncRNA using LC-MS/MS. Microbiol Spectr. 2023;11(3):e0356422. doi: 10.1128/spectrum.03564-22 37036375 PMC10269570

[21] Holmes MJ, Padgett LR, Bastos MS, Sullivan WJ, Jr. m6A RNA methylation facilitates pre-mRNA 3’-end formation and is essential for viability of Toxoplasma gondii. PLoS Pathog. 2021;17(7):e1009335. doi: 10.1371/journal.ppat.1009335 34324585 PMC8354455

[22] Nakamoto MA, Lovejoy AF, Cygan AM, Boothroyd JC. mRNA pseudouridylation affects RNA metabolism in the parasite Toxoplasma gondii. RNA. 2017;23(12):1834–49. doi: 10.1261/rna.062794.117 28851751 PMC5689004

[23] Hu RS, He JJ, Elsheikha HM, Zhang FK, Zou Y, Zhao GH, et al. Differential brain MicroRNA expression profiles after acute and chronic infection of mice with Toxoplasma gondii oocysts. Front Microbiol. 2018;9:2316. doi: 10.3389/fmicb.2018.02316 30333806 PMC6176049

[24] Zhang Y, Zhang X, Shi J, Tuorto F, Li X, Liu Y, et al. Dnmt2 mediates intergenerational transmission of paternally acquired metabolic disorders through sperm small non-coding RNAs. Nat Cell Biol. 2018;20(5):535–40. doi: 10.1038/s41556-018-0087-2 29695786 PMC5926820

[25] Guo H, Xia L, Wang W, Xu W, Shen X, Wu X, et al. Hypoxia induces alterations in tRNA modifications involved in translational control. BMC Biol. 2023;21(1):39. doi: 10.1186/s12915-023-01537-x 36803965 PMC9942361

[26] Shi J, Zhang Y, Tan D, Zhang X, Yan M, Zhang Y, et al. PANDORA-seq expands the repertoire of regulatory small RNAs by overcoming RNA modifications. Nat Cell Biol. 2021;23(4):424–36. doi: 10.1038/s41556-021-00652-7 33820973 PMC8236090

[27] He T, Guo H, Shen X, Wu X, Xia L, Jiang X, et al. Hypoxia-induced alteration of RNA modifications in the mouse testis and spermdagger. Biol Reprod. 2021;105(5):1171–8. 34296257 10.1093/biolre/ioab142

[28] Guo H, Shen X, Hu H, Zhou P, He T, Xia L, et al. Alteration of RNA modification signature in human sperm correlates with sperm motility. Mol Hum Reprod. 2022;28(9). doi: 10.1093/molehr/gaac031 35959987 PMC9422301

[29] He T, Guo H, Xia L, Shen X, Huang Y, Wu X, et al. Alterations of RNA modification in mouse germ cell-2 spermatids under hypoxic stress. Front Mol Biosci. 2022;9:871737. doi: 10.3389/fmolb.2022.871737 35775084 PMC9237606

[30] Richter F, Plehn JE, Bessler L, Hertler J, Jorg M, Cirzi C, et al. RNA marker modifications reveal the necessity for rigorous preparation protocols to avoid artifacts in epitranscriptomic analysis. Nucleic Acids Res. 2022;50(8):4201–15. doi: 10.1093/nar/gkab1150 34850949 PMC9071408

[31] Nguyen TD, Bigaignon G, Van Broeck J, Vercammen M, Nguyen TN, Delmee M, et al. Acute and chronic phases of Toxoplasma gondii infection in mice modulate the host immune responses. Infect Immun. 1998;66(6):2991–5. doi: 10.1128/IAI.66.6.2991-2995.1998 9596779 PMC108301

[32] Hester J, Mullins J, Sa Q, Payne L, Mercier C, Cesbron-Delauw MF, et al. Toxoplasma gondii antigens recognized by IgG antibodies differ between mice with and without active proliferation of tachyzoites in the brain during the chronic stage of infection. Infect Immun. 2012;80(10):3611–20. doi: 10.1128/IAI.00604-12 22851753 PMC3457563

[33] Sana M, Rashid M, Rashid I, Akbar H, Gomez-Marin JE, Dimier-Poisson I. Immune response against toxoplasmosis-some recent updates RH: Toxoplasma gondii immune response. Int J Immunopathol Pharmacol. 2022;36:3946320221078436. doi: 10.1177/03946320221078436 35227108 PMC8891885

[34] Hwang YS, Shin JH, Yang JP, Jung BK, Lee SH, Shin EH. Characteristics of infection immunity regulated by Toxoplasma gondii to maintain chronic infection in the brain. Front Immunol. 2018;9:158. doi: 10.3389/fimmu.2018.00158 29459868 PMC5807351

[35] Zhang Y, Zhang LS, Dai Q, Chen P, Lu M, Kairis EL, et al. 5-methylcytosine (m(5)C) RNA modification controls the innate immune response to virus infection by regulating type I interferons. Proc Natl Acad Sci U S A. 2022;119(42):e2123338119. doi: 10.1073/pnas.2123338119 36240321 PMC9586267

[36] Wang H, Hu X, Huang M, Liu J, Gu Y, Ma L, et al. Mettl3-mediated mRNA m(6)A methylation promotes dendritic cell activation. Nat Commun. 2019;10(1):1898. doi: 10.1038/s41467-019-09903-6 31015515 PMC6478715

[37] Song H, Song J, Cheng M, Zheng M, Wang T, Tian S, et al. METTL3-mediated m(6)A RNA methylation promotes the anti-tumour immunity of natural killer cells. Nat Commun. 2021;12(1):5522. doi: 10.1038/s41467-021-25803-0 34535671 PMC8448775

[38] Elsabbagh RA, Rady M, Watzl C, Abou-Aisha K, Gad MZ. Impact of N6-methyladenosine (m(6)A) modification on immunity. Cell Commun Signal. 2022;20(1):140. doi: 10.1186/s12964-022-00939-8 36085064 PMC9461097

[39] Cui L, Ma R, Cai J, Guo C, Chen Z, Yao L, et al. RNA modifications: importance in immune cell biology and related diseases. Signal Transduct Target Ther. 2022;7(1):334. doi: 10.1038/s41392-022-01175-9 36138023 PMC9499983

[40] Guzzi N, Ciesla M, Ngoc PCT, Lang S, Arora S, Dimitriou M, et al. Pseudouridylation of tRNA-derived fragments steers translational control in stem cells. Cell. 2018;173(5):1204–16 e26. doi: 10.1016/j.cell.2018.03.008 29628141

[41] Pandey KK, Madhry D, Ravi Kumar YS, Malvankar S, Sapra L, Srivastava RK, et al. Regulatory roles of tRNA-derived RNA fragments in human pathophysiology. Mol Ther Nucleic Acids. 2021;26:161–73. doi: 10.1016/j.omtn.2021.06.023 34513302 PMC8413677

[42] Obregon-Henao A, Duque-Correa MA, Rojas M, Garcia LF, Brennan PJ, Ortiz BL, et al. Stable extracellular RNA fragments of Mycobacterium tuberculosis induce early apoptosis in human monocytes via a caspase-8 dependent mechanism. PLoS One. 2012;7(1):e29970. doi: 10.1371/journal.pone.0029970 22253841 PMC3253812

[43] Keegan C, Krutzik S, Schenk M, Scumpia PO, Lu J, Pang YLJ, et al. Mycobacterium tuberculosis transfer RNA Induces IL-12p70 via synergistic activation of pattern recognition receptors within a cell network. J Immunol. 2018;200(9):3244–58. doi: 10.4049/jimmunol.1701733 29610140 PMC5916334

