# Supplementary figures and images for: Altered landscape of total RNA, tRNA and sncRNA modifications in the liver and spleen of mice infected by Toxoplasma gondii
Source: PLoS Negl Trop Dis. 2024 Jun 21;18(6):e0012281. doi: 10.1371/journal.pntd.0012281 (PMC11221703; doi:10.1371/journal.pntd.0012281)

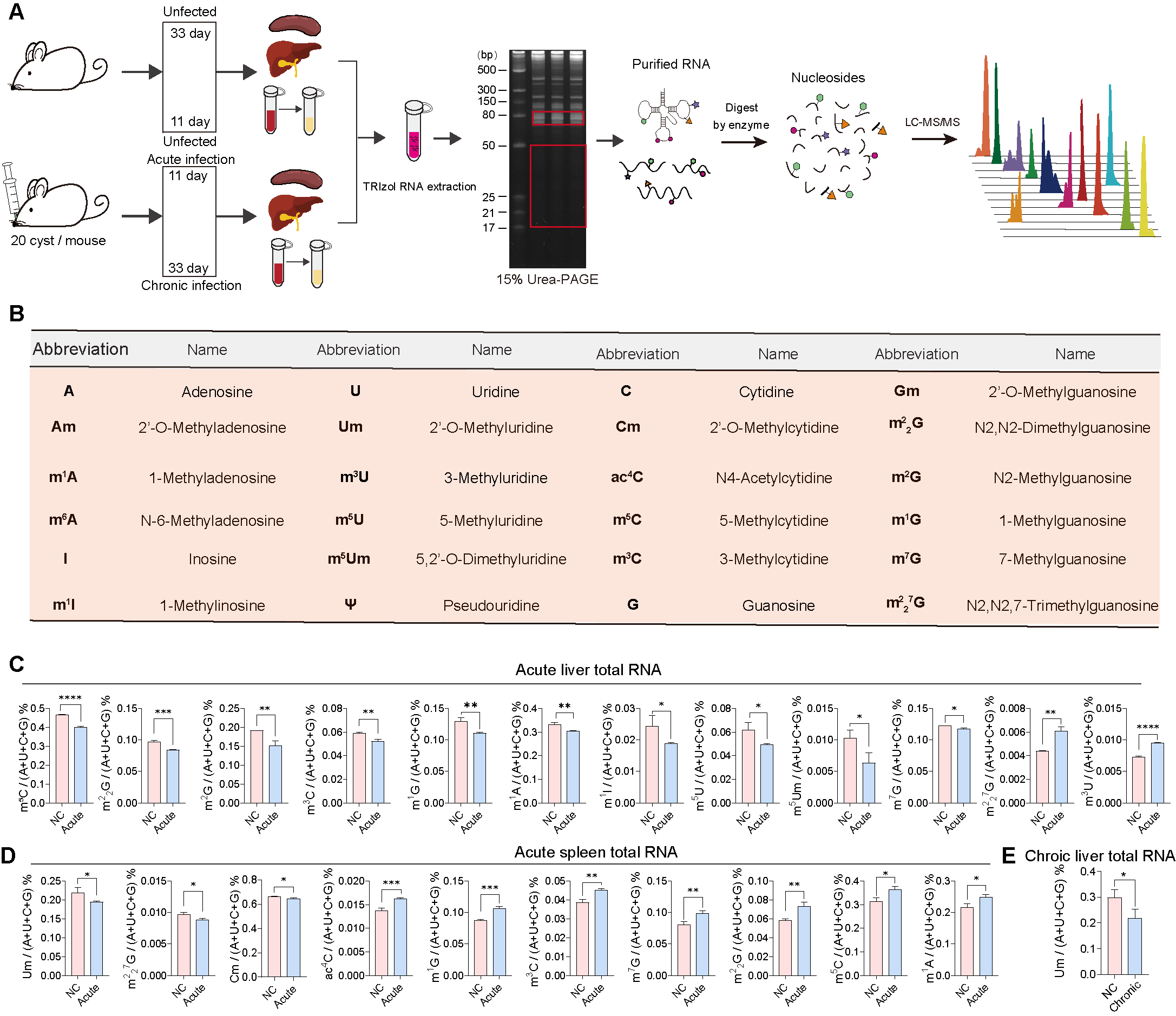

Supplement: S1 Fig — (A) A schematic illustration depicting the experimental procedures used for detecting and quantifying RNA modifications in the spleen, liver, and serum of mice. (B) List of the used nucleobase standards. (C) Comparison of RNA modifications in total RNA of the liver between the control group and the acute infection group. (D) Comparison of RNA modifications in total RNA of the spleen between the control group and the acute infection group. (E) Comparison of RNA modifications in the total RNA of liver between the control group and the chronic infection group. All results are shown as mean ± SEM based on three biological replicates. Statistical analysis was conducted using unpaired Student’s t-test. Significant differences are indicated by asterisk as follows: *p < 0.05, **p < 0.01, ***p < 0.001, ****p < 0.0001. (TIF) [file pntd.0012281.s001.tif]

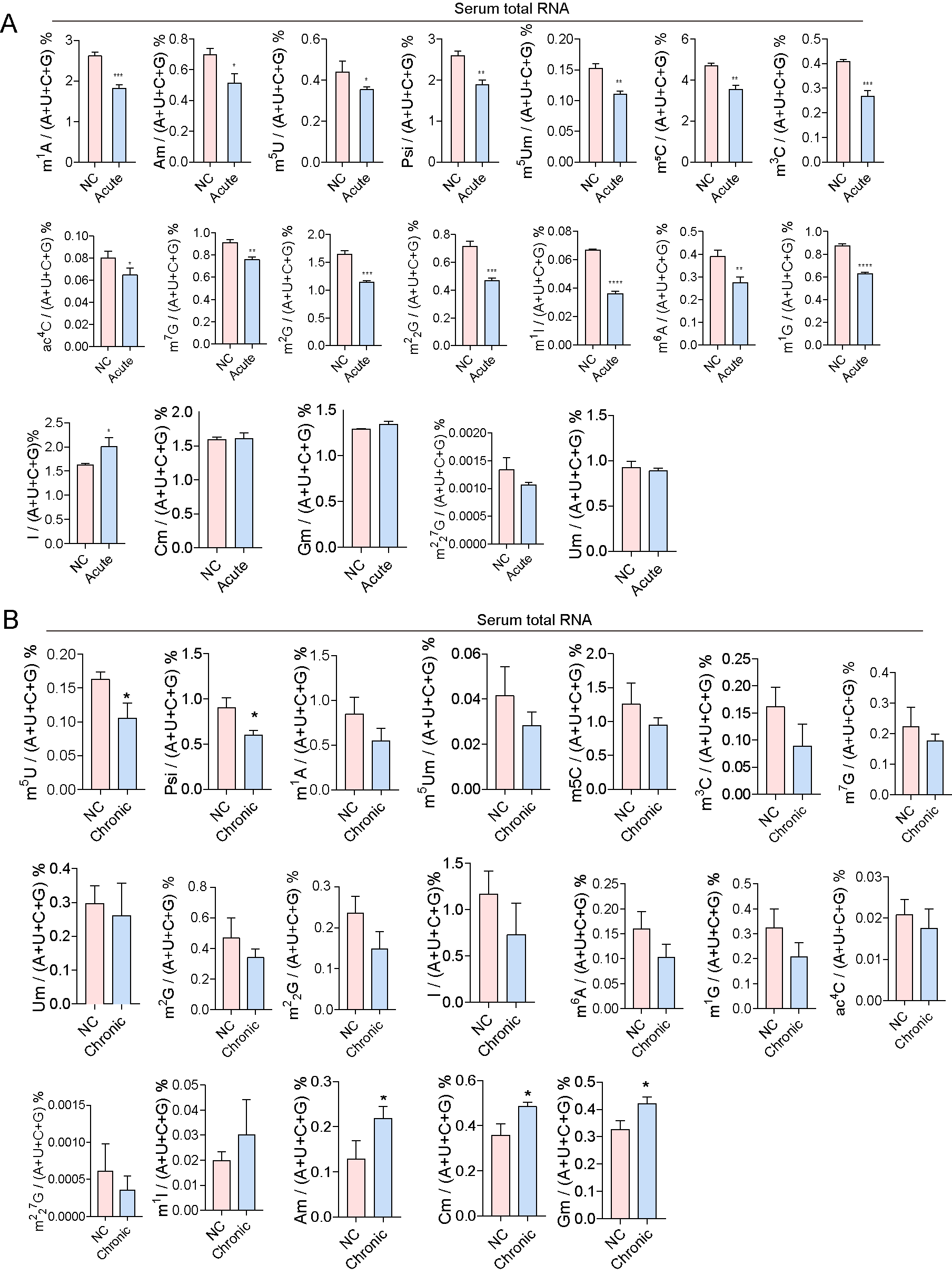

Supplement: S2 Fig — (A) Comparison of RNA modifications in the serum total RNA between the control group and the acute infection group. (B) Comparison of RNA modifications in the serum total RNA between the control group and the chronic infection group. All results are shown as mean ± SEM based on biological triplicate. Statistical analysis was conducted using unpaired Student’s t-test. Significant differences are indicated by asterisk as follows: *p < 0.05, **p < 0.01, ***p < 0.001, ****p < 0.0001. (TIF) [file pntd.0012281.s002.tif]
